# Compositional Changes and Comparative Analysis of Oral Microbial Community During the Formation of Canine Dental Calculus

**DOI:** 10.3390/ani15223335

**Published:** 2025-11-19

**Authors:** Liwei Zeng, Lei Shi, Yufei Yang, Dongqiang Zheng, Wenkai Zhang, Jingyi Yang, Meilin Qiao, Hao Shi

**Affiliations:** 1 College of Veterinary Medicine, China Agricultural University, Beijing 100193, China; liweizeng@cau.edu.cn (L.Z.);; 2 China Agricultural University Veterinary Teaching Hospital, Beijing 100193, China

**Keywords:** dog, dental calculus, oral flora, biomarker, 16S rRNA gene, PacBio

## Abstract

Periodontal disease remains a prevalent clinical condition in dogs, significantly impacting the quality of life of affected animals. Dental calculus is closely associated with periodontal disease, primarily because it serves as a reservoir for dental plaque and bacterial toxins, that contribute to gingival inflammation. To investigate the changes in the oral microbiome during the formation of dental calculus, a total of 30 dogs were enrolled and divided into three groups: healthy dogs (H, *n* = 10), provided tooth surface samples; dogs with dental plaque (P, *n* = 10) supplied plaque samples; and dogs with dental calculus (C, *n* = 10) provided calculus samples. DNA was subsequently extracted from all collected samples and subjected to PacBio sequencing. The results revealed significant differences in the composition and diversity of the oral microbiome between the plaque and calculus groups compared to the healthy group. As calculus gradually formed, the relative abundance of Pseudomonadota decreased significantly, while the proportions of Bacillota, Actinomycetota, Campylobacterota, and Thermodesulfobacteriota increased markedly. *Bacteroides pyogenes* and *Peptostreptococcus canis* were identified as potential biomarkers associated with calculus formation, whereas *Conchiformibius steedae* and *Pasteurellaceae bacterium canine oral taxon 271* may serve as indicators of healthy periodontal tissue. Functional prediction analysis revealed a notable reduction in the ABC transporter pathway in the oral microbiota of dogs with dental calculus compared to the healthy group. In conclusion, our study has revealed the compositional changes in the oral microbial community and potential metabolic pathways during the formation of dental calculus in dogs. These findings provide a theoretical foundation for the identification of potential microbial biomarkers and for developing preventive and therapeutic strategies for canine periodontal disease.

## Simple Summary

Dental calculus, a major contributor to canine periodontal disease, creates an ideal environment for oral bacteria to adhere to and colonize. However, the compositional changes and differences in the oral microbial community during the formation of canine dental calculus remain poorly characterized. Clarifying these microbial changes could enhance early diagnosis and treatment strategies for canine periodontal disease. In our study, we included 30 dogs, which were divided into three groups, and collected oral samples from each individual. We analyzed the bacterial composition of the oral microbiome through full-length 16S ribosomal RNA (rRNA) gene sequencing (V1–V9 regions). Our findings revealed significant differences in oral microbial community composition and predicted metabolic pathways between healthy dogs and those affected by plaque or calculus. Importantly, distinct taxonomic markers were identified at the phylum, genus, and species levels, which may serve as potential biomarkers for calculus formation and even periodontitis. However, the mechanistic roles of these species in calculus development require further investigation.

## 1. Introduction

Periodontal disease (PD) is one of the serious health issues affecting dogs and is often not detected until advanced stages. Diagnosis primarily relies on observable clinical signs, such as the color of periodontal tissues, the depth of periodontal pockets, bleeding upon probing, and radiographic examinations. However, these procedures are frequently poorly tolerated by animals and generally require anesthesia. Moreover, these diagnostic methods tend to provide information on tissue destruction that has already occurred. Therefore, it is crucial to develop quicker and less invasive approaches to assess the extent of dental calculus formation and current disease activity. Such advancements would enable earlier diagnosis of canine PD and improve predictions of its future progression [1,2,3]. The primary etiological factor in the onset and development of PD in dogs is the microbial community residing on the tooth surface, known as dental plaque [4,5]. Bacteria within this biofilm can be categorized into early or late colonizers. Early colonizers are typically facultative anaerobes and saccharolytic organisms, which utilize salivary mucoproteins and other glycoproteins as nutrient sources. In contrast, late colonizers are predominantly obligate anaerobes that primarily hydrolyze proteins. The progressive colonization of these bacteria facilitates the formation of biofilms, accompanied by a progressive increase in pathogenicity over time [6,7]. The accumulation of dental plaque leads to early-stage, reversible gingivitis. Under the combined influence of host factors and virulent bacterial activities, this condition can progress to late-stage, irreversible periodontitis. This progression involves bone and connective tissues destruction, periodontal pockets formation, gingival recession, and ultimately tooth loss [8]. This pathogenic process represents a complex interplay between the host’s defense mechanisms and the microbial community within plaque.

Dental calculus is regarded as a secondary etiological factor in PD. It is defined as calcified dental plaque covered by a non-mineralized bacterial layer and can be divided into supragingival and subgingival calculus. Supragingival calculus is commonly found on the tooth surfaces near the openings of salivary ducts, whereas subgingival calculus is randomly distributed throughout the oral cavity [9]. Canine dental calculus exhibits a distinct histological stratification: an intact bacterial community constitutes the surface layer; the central region is composed of a large amount of minerals, mucoid substances, cells, and bacterial fragments; the deeper layer adjacent to the tooth surface is primarily made up of leukocytes, desquamated epithelial cells, and intact bacteria [10]. The composition of dental calculus is dominated by inorganic minerals, principally calcium carbonate and calcium phosphate, as well as organic components such as proteins and carbohydrates [11]. The mineralization process involves the metabolic activities of bacterial colonies. During this process, bacterial biofilms, including bacterial species that may also be identified in saliva, accumulate within the mineralized deposits [11]. The mineralization process leading to the formation of dental calculus commences shortly after the formation of dental plaque [1]. Bacteria contribute to localized increases in calcium and phosphate concentrations, triggering biofilm mineralization and calculus development. Dental calculus mechanically irritates periodontal tissues. Moreover, its rough surface facilitates bacterial adhesion, thereby promoting further bacterial colonization and hindering the reattachment and proliferation of host cells [4,6].

The oral microbiota of dogs is highly complex ecosystem and is recognized as the second largest microbial community in the body, following the gut microbiota. Accurate identification of oral microorganisms at the species level is fundamental for analyzing the oral microbial community [12]. High-throughput sequencing of the 16S rRNA gene can reveal microbial community structure, phylogeny and abundance across diverse ecosystems [13,14]. Advances in sequencing technologies—particularly those targeting the 16S rRNA gene—have revolutionized microbiome research, making it central to understanding the biology and functional diversity of oral microbiota [12,15,16]. Sequencing of the 16S rRNA gene has long served as the gold standard for investigating the taxonomic composition of bacterial communities [17,18]. The first-generation Sanger sequencing technology has several limitations such as high sequencing costs, low throughput, and slow processing speed [19]. After 2005, the emergence of next-generation sequencing (NGS) technologies—such as Roche (Basel, Switzerland) 454 pyrosequencing, Illumina Solexa polymerase sequencing (San Diego, CA, USA), and Applied Biosystems (ABI, Waltham, MA, USA) SOLiD ligase sequencing—have improved sequencing accuracy. However, their taxonomic resolution remains limited, allowing analyses only at the genus level [20,21]. The third-generation sequencing (TGS) technologies, exemplified by Pacific Biosciences (PacBio) and Single Molecule Real-Time (SMRT), have further advanced the field. By increasing read length, these methods enable high-accuracy sequencing of the full-length 16S rRNA gene (V1–V9), thereby obtaining complete sequences from individual microorganisms. This provides greater taxonomic resolution and more accurate estimates of species richness and diversity [22,23,24]. Using the PacBio platform, circular consensus sequencing (CCS) generates highly accurate high-fidelity (HiFi) reads with self-correcting accuracy exceeding 99%. This facilitates microbial classification at the species level and allows comprehensive analyses of species composition, abundance, and community structure. Concurrently, the introduction of the DADA2 algorithm has enabled single-nucleotide resolution and near-zero error rates in full-length 16S rRNA gene analysis [25]. HiFi sequencing based on the PacBio platform has been widely applied to studies of oral, gut, and soil microbiomes. Previous research employing Pacific Biosciences (PacBio, Menlo Park, CA, USA) sequencing of human saliva, gingival crevicular fluid [26], and dental plaque samples [27] successfully generated full-length 16S rRNA sequences. These studies identified dominant bacterial species potentially associated with oral health or disease at the species level, thereby offering valuable insights for early diagnosis and disease prevention. Overall, the development of these sequencing technologies has substantially improved the ability to annotate novel microbial taxa and detect genetic variations within microbial communities.

Numerous studies have previously collected oral samples—including saliva, supragingival and subgingival plaque, and gingival crevicular fluid—from healthy dogs and those affected by gingivitis or periodontitis. These investigations employed bacterial culture, PCR, and 16S rRNA sequencing to explore the composition, diversity, and functional predictions of the canine oral microbiota in PD. However, the majority of these studies have predominantly focused on providing static composition of microbial communities. Furthermore, due to technical limitations associated with extracting bacterial DNA from mineralized dental calculus, few studies have characterized the bacterial composition within calculus. In this study, we collected multiple sample types: tooth surface specimens, dental plaque, and calculus. PacBio sequencing technology was employed to perform high-throughput sequencing of the full-length 16S rRNA genes from their microbial communities. This approach highlights variations in microbial composition among distinct oral ecological niches and reveals the compositional and functional succession patterns of oral microbiota during calculus formation—from free-floating states to biofilm formation and ultimately to mineralization. We further sought to bacterial taxa that may be selectively enriched during the mineralization process. Certain bacterial species present in dental calculus may play specific roles in calculus formation. Identifying such taxa could lead to the discovery of canine-specific microbial biomarkers of dental calculus formation. These findings are expected to provide theoretical foundations for early diagnosis of PD, development of canine oral probiotics, or targeted antimicrobial agents against mineralization-promoting microbiota.

## 2. Materials and Methods

### 2.1. Samples Collection and Eligibility Criteria

This study established a grading standard for canine periodontal health by comprehensively evaluating the gingival index (GI), plaque index (PI), and calculus index (CI) (Table 1) [28,29,30]. Based on these criteria, eligible cases were screened and categorized. The inclusion and exclusion criteria for sample selection were defined as follows.

The inclusion criteria for healthy individuals were as follows: (1) clinically healthy gingiva with no visible signs of inflammation along the gingival margin, normal periodontal pocket (free gingival) depth ≤ 3 mm, bleeding on probing (BOP) ≤ 5%, and no macroscopically visible plaque or calculus on tooth surfaces; (2) absence of oral abscesses; (3) no presence of premalignant or malignant lesions; (4) no candidiasis. Dogs with plaque accumulation were included if they met the following: BOP-positive sites ≥ 10%, GI = 0.5–2, PI = 0.5–2.5, and no clinical attachment loss [31]. Dogs with calculus were included if their CI was ≥2.

Exclusion criteria for all subjects included: (1) presence of any systemic disease or condition that could interfere with study results (e.g., diabetes, pregnancy, immune system disorders, etc.) [31]; (2) administration of systemic antibiotics, anti-inflammatory drugs, or probiotics preparations within 4 weeks prior to sampling.

Finally, 10 healthy dogs (H), 10 plaque-affected dogs (P), and 10 calculus-affected dogs (C) were selected (Table 2). No statistically significant differences in body weight and sex were observed among the groups. Tooth surface samples were collected from healthy dogs, while plaque and calculus samples were obtained from the respective affected groups under general anesthesia. For each enrolled dog, detailed information was recorded, including breed, age, sex, weight, GI, PI, CI, diet, sampling site (identified using the modified Triadan tooth numbering system), previous medication, and the time since last treatment or cleaning (Supplementary Table S1).

Prior to sampling, the target tooth surfaces were rinsed with sterile saline solution to remove saliva and food debris, followed by drying of the sampling sites. In healthy dogs, tooth surface specimens were collected by swabbing the tooth surfaces with flocked swabs. The tips of two swabs were then aseptically cut off and placed into sterile EP tubes. For dogs with dental plaque, supragingival plaque was carefully scraped using a dental curette, ensuring that any samples mixed with blood were discarded. The collected plaque was then transferred onto flocked swabs, and the swab tips were trimmed and place into sterile EP tubes. For dogs with dental calculus, visible calculus and deposits were obtained from the tooth surfaces using calculus forceps, again discarding any samples showing signs of bleeding. The acquired calculus was placed directly into sterile EP tubes.

All samples were sealed immediately in zip-lock bags and frozen immediately on dry ice, and kept at −80 °C before further processing.

### 2.2. DNA Extraction, PCR Amplification and PacBio Sequencing

According to the kit instructions, bacterial genomic DNA was extracted from canine oral samples. The DNA concentration and purity were measured using NanoDrop 2000 spectrophotometer (Thermo Scientific, Waltham, MA, USA), and DNA integrity was verified by 1% agarose gel electrophoresis. The DNA concentration of each sample is provided in Supplementary Table S2.

The V1–V9 hypervariable regions of the 16S rRNA gene were amplified using the bacterial primers 27F (AGRGTTYGATYMTGGCTCAG) and 1492R (RGYTACCTTGTTACGACTT). Amplification reactions (20 μL volume) consisted of 2 × Pro Taq 10 μL, forward primer (5 μM) 0.8 μL, reverse primer (5 μM) 0.8 μL, template DNA 10 ng/μL and double-distilled water. The PCR amplification was performed as follows: initial denaturation at 95 °C for 3 min, followed by 29 cycles of denaturing at 95 °C for 30 s, annealing at 60 °C for 30 s and extension at 72 °C for 45 s, and single extension at 72 °C for 10 min, and end at 10 °C. The PCR products were analyzed via 2% agarose gel electrophoresis, and target bands were excised and purified using the AxyPrep DNA Gel Extraction Kit (AXYGEN, Union, CA, USA). The purified products were quantified using Qubit 4.0 Fluorometer (Thermo Fisher Scientific, Waltham, MA, USA). Subsequently, the amplicons were pooled in equimolar amounts according to the sequencing requirements per sample.

Library preparation was constructed using the SMRTbell Prep Kit 3.0 (Pacifc Biosciences, Menlo Park, CA, USA), which included the following steps: (1) DNA damage repair, (2) end repair, and (3) adapter ligation. Sequencing was performed on the PacBio Sequel IIe System (Pacifc Biosciences, Menlo Park, CA, USA) by Majorbio Bio-Pharm Technology Co., Ltd. (Shanghai, China), and approximately 1500 bp was specified as the insert size. The sequencing results were considered as raw data.

### 2.3. Statistical Analysis

CCS mode in SMRT Link v11.0 were used to perform multiple sequencing passes, alignment, and error correction of the raw reads, thereby generating high-quality CCS reads [25]. Based on barcode information, the lima software (v2.12.0) was employed to demultiplex the reads and obtain data for each sample. Primer removal and sequence orientation were standardized using the DADA2_CCS plugin within the QIIME2 pipeline (parameters for DADA2: maxEE = 5, truncQ = 0), which filters low-quality reads according to expected error rates. These steps collectively eliminated a large proportion of unreliable or irrelevant sequences, resulting in quality-controlled data. Subsequently, the DADA2 algorithm learned error model from the specific dataset and performed sample inference. This process reduced sequence redundancy and corrected sequencing errors, while also identified and removed chimeric sequences. The chimeric sequences removal method adopted was consensus, with the consensus mode set to “molecule”. The output was a set of accurate, non-chimeric amplicon sequence variants (ASVs). The resulting dataset was denoised data. Taxonomic classification of ASVs was performed using the BLAST classifier in QIIME2 against the NT_Taxon_core_v2024/16s_bacteria database (version 138).

Alpha diversity, including the Chao1 and Shannon index, was calculated using the mothur software (v1.30.2). Differences in alpha diversity across groups were assessed using the Kruskal–Wallis H test. Beta diversity was evaluated through Principal Co-ordinates Analysis (PCoA) based on unweighted UniFrac distance to evaluate similarities in microbial community structure among sample groups. The significance of intergroup differences was determined using ANOSIM analysis. Linear Discriminant Analysis Effect Size (LEfSe) was employed to identify taxa with significant abundance differences among groups, from phylum to species level. This analysis employed the nonparametric Kruskal–Wallis sum-rank test to detect significant differences in species abundance among groups. Species showing significant variations were further examined using the (unpaired) Wilcoxon rank-sum test to assess the consistency of these differences across subgroup comparisons. Finally, Linear Discriminant Analysis (LDA) was applied to estimate the extent to which each species’ abundance contributed to the effect of differences (LDA score). A higher LDA score indicates a greater difference in species between groups. Functional profiling of microbial communities was performed using PICRUSt2 (v2.2.0), which predicted the metabolic potential based on 16S rRNA gene data. This analysis provided an overview of the functional attributes of microbial communities across samples and may allow the identification of potential microbial functions associated with disease progression. During the multiple comparisons of microbial taxonomic abundances, multiple testing correction was performed using the false discovery rate (FDR) to adjust *p*-values. This approach minimized false positives and enhanced the reliability of statistical inferences, facilitating the accurate identification of microbial taxa significantly associated with dental calculus formation.

## 3. Results

### 3.1. Sequence Analysis

Following denoising, the 30 samples yielded a total of 761,463 sequences, with the number of sequences per sample ranging from 12,129 to 62,700. Since identical ASVs could be present across samples, duplicate ASVs were removed, resulting in 5153 unique ASVs (Supplementary Table S3). To minimize the potential influence of sequencing depth on subsequent analyses of alpha and beta diversity, the denoising sequences were rarefied to the minimum sequence number (12,129), standardizing all samples to 12,129 sequences. After rarefaction and duplicate removal, 5102 ASVs were retained. These optimized sequences were employed for subsequent taxonomic analyses.

**Figure 1:**
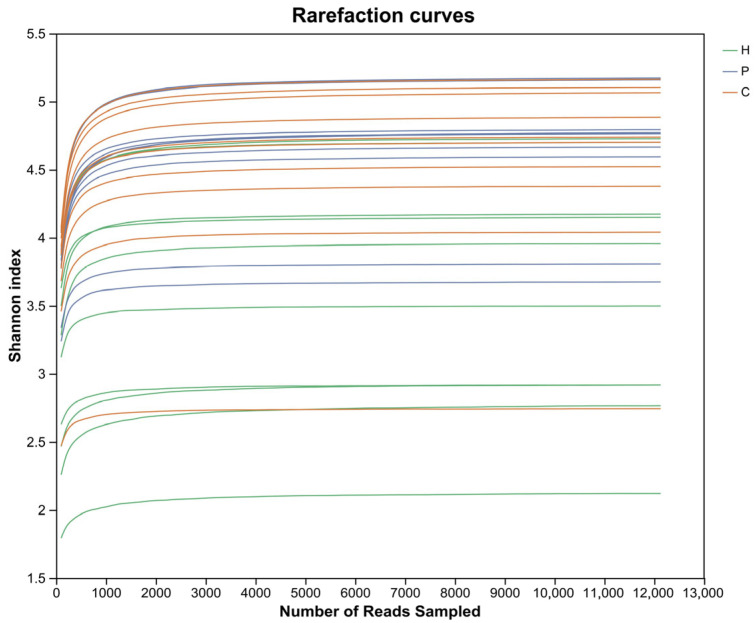
Rarefaction curves. Lines in different colors represent distinct sample groups. The *x*-axis denotes the randomly sampled sequencing data volume, and the *y*-axis indicates the observed Shannon index. As the number of sequences increases, the rarefaction curves for all samples approach a saturating plateau, demonstrating that the rationality of the data and its suitability for subsequent bioinformatics analyses. The number of ASVs sequenced across all three groups is sufficient to ensure the accuracy of the results. H represents healthy dogs; P represents dogs with dental plaque; C represents dogs with dental calculus.

Rarefaction curves for each sample showing that the increase in Shannon index—reflecting community diversity—gradually diminished as the number of reads sampled increased, with the curves eventually plateauing (Figure 1). This indicates that the sequencing data volume employed in this study was reasonable and sufficiently representative of community diversity.

### 3.2. Analysis of Microbial Diversity During Canine Dental Calculus Formation

**Figure 2:**
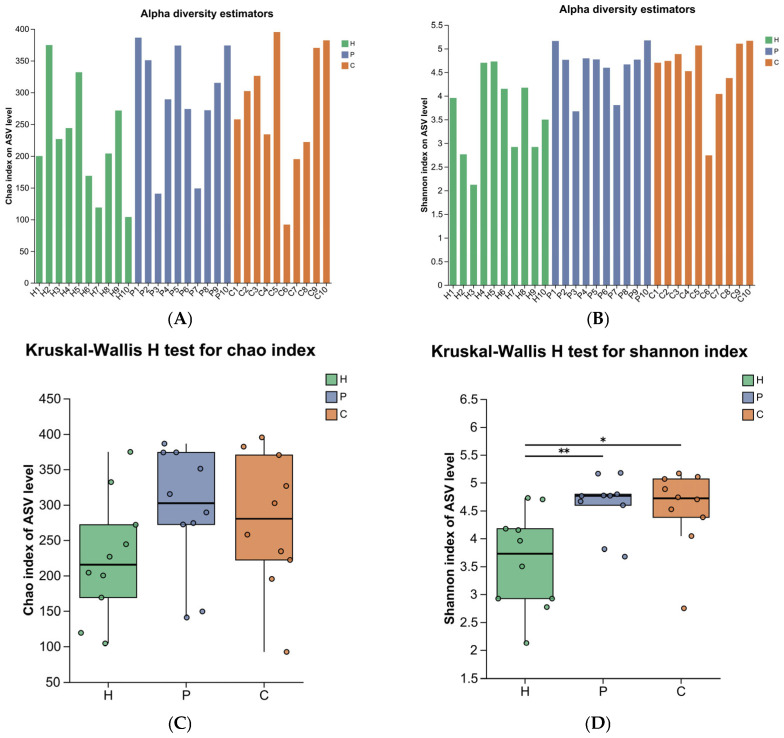
Alpha diversity analysis of microbial species in the oral cavity of the three groups. (**A**) Boxplots of microbiome Chao1 index. (**B**) Boxplots of microbiome Shannon index. (**C**) Kruskal–Wallis H test for significant differences in Chao1 index. (**D**) Kruskal–Wallis H test for significant differences in Shannon index. (* denotes 0.01 < *p* < 0.05, ** denotes 0.001 < *p* ≤ 0.01).

To assess the richness and diversity of the oral microbial community during the development of canine dental calculus, 16S rRNA sequencing was performed, and alpha diversity was evaluated using the Chao1 index and Shannon index. The Chao1 index showed no significant differences among the three groups (*p* > 0.05) (Figure 2A,C). However, the Shannon index revealed statistically significant differences between the healthy group and both the plaque and calculus groups (*p* < 0.05) (Figure 2B,D).

**Figure 3:**
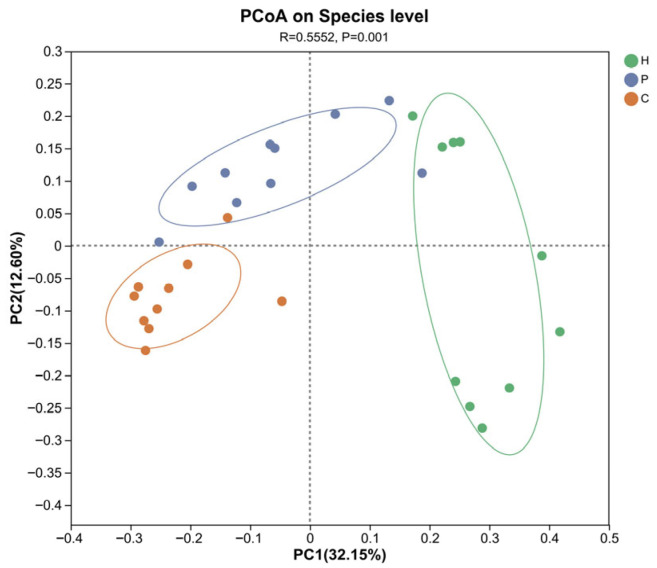
Beta diversity analysis of microbial species in the oral cavity of the samples. PCoA based on unweighted unifrac. ANOSIM was employed to detect the significance of intergroup differences. The *x*-axis and *y*-axis represent two selected principal coordinate components, with the percentages indicating the proportion of variation in sample composition explained by each coordinate. The scales on the axes denote relative distances and carry no intrinsic meaning. Points of different colors denote samples from distinct groups. The closer two sample points are, the more similar their oral microbial compositions.

Beta diversity analysis was assessed using unweighted unifrac distance algorithm and Principal Coordinate Analysis (PCoA), while differences between groups were tested with ANOSIM. These analyses could assess the similarity or dissimilarity in microbial community composition across samples. The results show that there are statistically significant differences in the community composition among the healthy, dental plaque, and dental calculus dogs (*p* < 0.05). The first two principal coordinate components account for 29.15% and 11.53% of the total variation, respectively (Figure 3).

### 3.3. Analysis of Species Composition and Differences in the Microbiota During Canine Dental Calculus Formation

**Figure 4:**
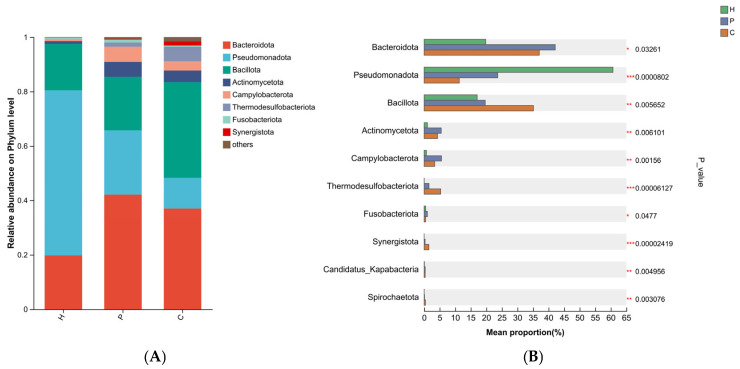
Phylum-level microbial composition of the samples. Taxa with a relative abundance below 1% were grouped as “others”. (**A**) Relative abundance of bacterial phyla among each group. (**B**) The Kruskal–Wallis H test was used to assess the significance of differences in oral microbial abundance at the phylum level. (* denotes 0.01< *p* < 0.05, ** denotes 0.001 < *p* ≤ 0.01, *** denotes *p* ≤ 0.001).

The composition of the oral bacterial communities at the phylum, genus, and species levels in the healthy, dental plaque, and dental calculus groups is shown in the bar plot in Figure 3. The results revealed that at the phylum level, the dominant taxa across all three groups included Bacteroidota (19.79%, 42.13%, and 37.01%, respectively), Pseudomonadota (60.68%, 23.65%, and 11.30%), and Bacillota (17.04%, 19.63%, and 35.13%), which together accounted for over 97%, 85%, and 83% of the total relative abundance in the healthy, plaque, and calculus groups, respectively (Figure 4A). Two phyla, Bacillota and Thermodesulfobacteriota, were significantly enriched in the calculus group. Pseudomonadota was most abundant in the healthy group, while Bacteroidota, Actinomycetota, and Campylobacterota were predominant in the plaque group (Figure 4B).

**Figure 5:**
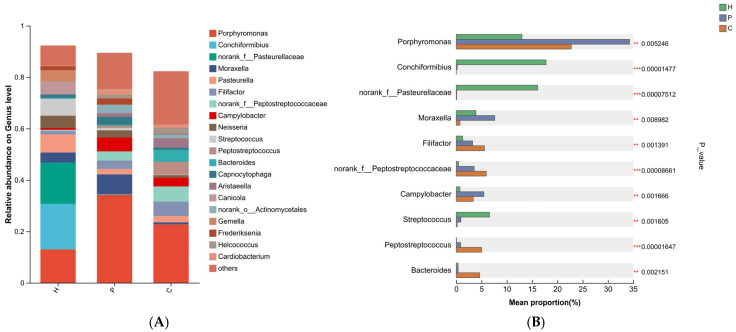
Genus-level microbial composition of the samples. Taxa with a relative abundance below 1% were grouped as “others”. (**A**) Relative abundance of bacterial genus among each group. (**B**) The Kruskal–Wallis H test was used to assess the significance of differences in oral microbial abundance at the genus level. (** denotes 0.001 < *p* ≤ 0.01, *** denotes *p* ≤ 0.001).

At the genus level, the predominant bacterial genus across the three sample groups was *Porphyromonas* (12.99%, 34.26%, and 22.74%, respectively) (Figure 5A). *Conchiformibius* and *Pasteurellaceae* were the dominant genera in the healthy group. In the plaque group, three bacterial taxa were significantly enriched: *Porphyromonas*, *Moraxella*, and *Campylobacter*. Compared to the healthy and plaque groups, *Peptostreptococcaceae*, *Filifactor*, and *Peptostreptococcus* exhibited the highest prevalence in the dental calculus group (Figure 5B).

**Figure 6:**
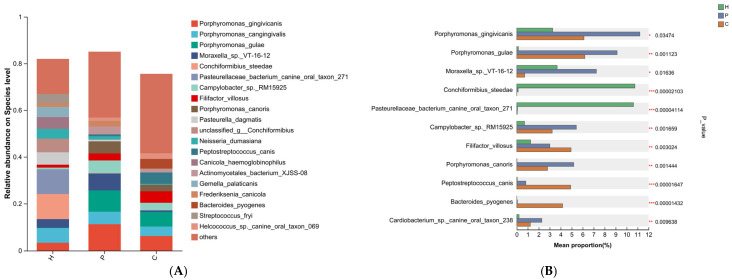
Species-level microbial composition of the samples. Taxa with a relative abundance below 1% were grouped as “others”. (**A**) Relative abundance of bacterial species among each group. (**B**) The Kruskal–Wallis H test was used to assess the significance of differences in oral microbial abundance at the species level. (* denotes 0.01 < *p* < 0.05, ** denotes 0.001 < *p* ≤ 0.01, *** denotes *p* ≤ 0.001).

At the species level, the healthy group was dominated by *Conchiformibius steedae* (10.75%) and *Pasteurellaceae bacterium canine oral taxon 271* (10.62%). In the plaque group, five species were notably predominant: *Porphyromonas gingivicanis* (11.20%), *Porphyromonas gulae* (9.13%), *Moraxella* sp. *VT-16-12* (7.24%), *Campylobacter* sp. *RM15925* (5.42%), and *Porphyromonas canoris* (5.18%). The dental calculus group exhibited the highest abundances of *Filifactor villosus* (4.94%), *Peptostreptococcus canis* (4.90%), *Bacteroides pyogenes* (4.17%), and *Desulfomicrobium orale* (2.80%) (Figure 6A,B).

**Figure 7:**
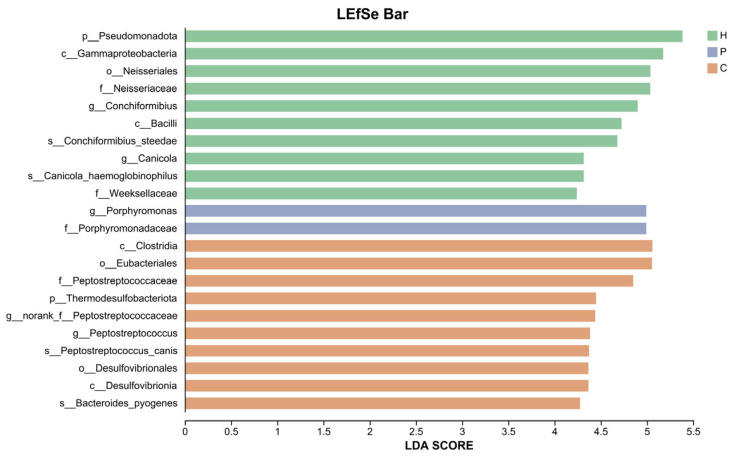
LEfSe analysis of oral microbial species in the samples. The *y*-axis denotes taxa exhibiting significant differences between the H, P and C groups, while the *x*-axis represents the score value for each taxon in the LDA. The all-against-all comparison strategy (more strict) is employed, whereby a species is only considered distinct if it exhibits differences across multiple groups. Only taxa with LDA score > 4 are displayed.

Using LEfSe analysis, we identified and compared characteristic differential taxa within the oral microbiota across the three sample groups. LDA scores were obtained via LDA, where higher scores indicate a greater contribution of the species abundance to the observed differences. The results are presented in Figure 7. Based on LDA score > 4, we identified the differentially abundant species across the three groups. The H group was characterized by the dominant phylum Pseudomonadota, the predominant genus *Conchiformibius*, and the key species *Conchiformibius steedaes* and *Canicola haemoglobinophilus*. In the P group, the genus *Porphyromonas* was identified as a signature taxon. The C group exhibited Thermodesulfobacteriota as the most discriminative phylum, with *Peptostreptococcaceae* and *Peptostreptococcus* as the dominant genera, and *Peptostreptococcus canis* and *Bacteroides pyogenes* as the major species. In conclusion, significant differences in oral microbial composition were observed among the H, P, and C groups, each harboring distinctive microbial signatures.

### 3.4. Differential Functional Analysis of Microbial Communities During Canine Dental Calculus Formation

**Figure 8:**
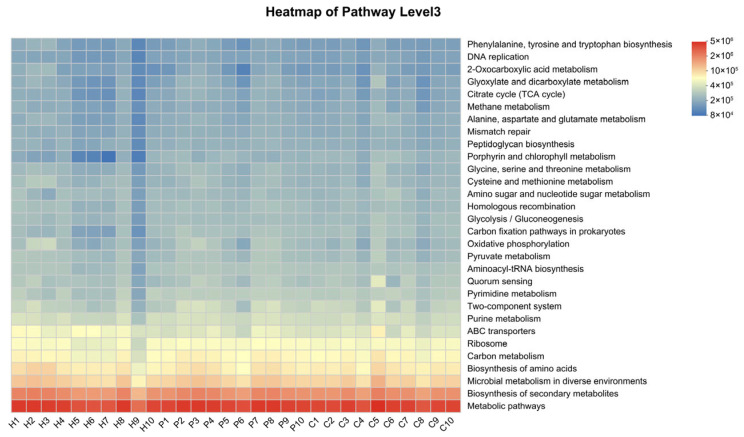
The heatmap of the top 20 metabolic pathway level III of each group. The *x*-axis indicates group and sample identification numbers, while the *y*-axis represents the names of metabolic pathway level III. Variation in functional abundance across samples is represented by a gradient color scale, with the legend indicating the numerical values associated with the color gradient.

To investigate how oral microbiota influence dental calculus formation, we subsequently applied PICRUSt2 to map the sequencing data from all samples across the three groups to the Kyoto Encyclopedia of Genes and Genomes (KEGG) database. This allowed us to identify metabolic functions associated with different genes and ultimately calculate the relative abundance of various metabolic pathways within the samples. At level III metabolic pathway, the functional genes across all three groups were primarily enriched in metabolic pathways, biosynthesis of secondary metabolites, microbial metabolism in diverse environments, biosynthesis of amino acids, and carbon metabolism (Figure 8).

**Figure 9:**
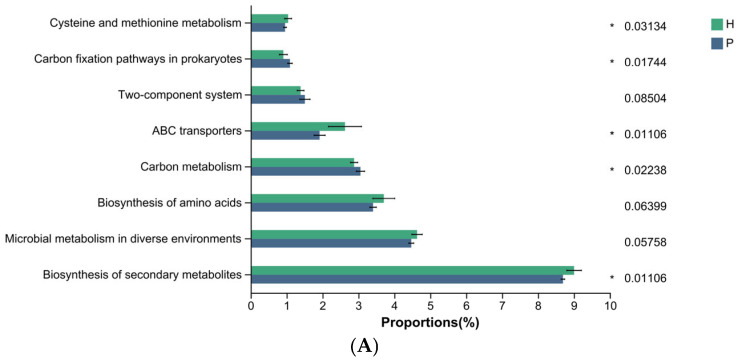

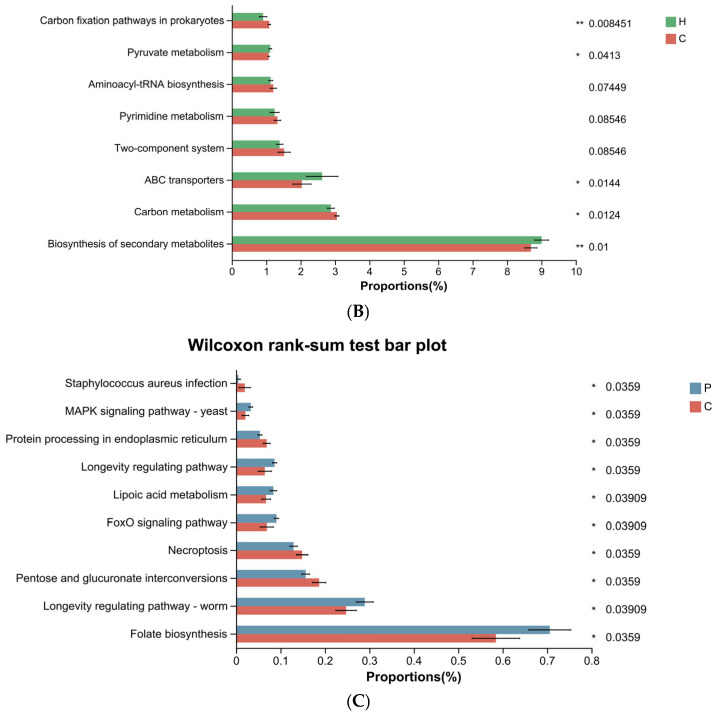
The three bar plots show metabolic pathway level III that exhibits significant intergroup differences. (**A**) H vs. P; (**B**) H vs. C; (**C**) P vs. C. (* denotes 0.01 < *p* < 0.05, ** denotes 0.001 < *p* ≤ 0.01).

Using the Wilcoxon rank-sum test, we identified differentially enriched metabolic pathways between groups. Compared to the H group, the P group showed increased relative abundance in two metabolic pathways: carbon fixation pathways in prokaryotes and carbon metabolism (Figure 9A). In contrast, the C group exhibited a decrease in ABC transporters and biosynthesis of secondary metabolites, while pathways such as carbon fixation pathways in prokaryotes and carbon metabolism were enriched relative to the H group (Figure 9B). Furthermore, compared to the P group, the C group demonstrated increased relative abundance in four metabolic pathways: staphylococcus aureus infection, protein processing in endoplasmic reticulum, necroptosis, and pentose and glucuronate interconversions. Conversely, pathways including MAPK signaling pathway—yeast, longevity regulating pathway, lipoic acid metabolism, FoxO signaling pathway, longevity regulating pathway—worm, and folate biosynthesis were significantly reduced (Figure 9C).

## 4. Discussion

This study revealed that, compared to healthy dogs, there was no significant difference in the Chao1 richness index in dogs with dental plaque or calculus; however, the Shannon diversity index was notably elevated. While the Chao1 index reflects only species richness, the Shannon index incorporates both richness and evenness. Thus, it can be inferred that the oral microbial communities of dogs with plaque and calculus showed no significant change in species richness (i.e., total number of species) compared to the healthy group, but community evenness was markedly enhanced. This suggests a reduction in the relative abundance of dominant health-associated species and an increase in less abundant species, ultimately leading to a significant rise in overall species diversity during calculus formation. Furthermore, combined with the beta diversity analysis—specifically, the PCoA—the compositions of the oral microbial community also underwent significant alterations in dogs with plaque and calculus. In conclusion, compared to healthy dogs, those with dental plaque and calculus exhibit significant changes in both the composition and diversity of their oral microbiota, providing compelling evidence that PD is associated with oral microbial dysbiosis.

In healthy states, the oral microbiome is dominated by Gram-negative bacteria (Bacteroidota and Pseudomonadota). As dental plaque biofilm matures and dental calculus forms, the proportion of Gram-negative bacteria gradually decreases, while that of Gram-positive bacteria (Bacillota, Actinomycetota) progressively increases. These Gram-positive bacteria are capable of secreting greater amounts of extracellular polysaccharides and proteins, thereby promoting biofilm formation and stability. This shift may also be attributed to the increasingly anaerobic environment, which facilitates enhanced anaerobic glycolysis and fermentation processes [32]. Even as the condition of PD deteriorates and the proportion of Gram-positive bacteria increases, Gram-negative bacteria still dominate the oral microbial community. That is, both healthy and periodontally diseased canine oral microbiomes are primarily composed of Gram-negative bacteria. However, other studies have reported that the oral flora of healthy dogs is dominated by Gram-negative bacteria, while that of dogs with PD is predominantly Gram-positive [33,34,35]. These discrepancies may arise from differences in sampling methods, sampling sites, and microbial detection techniques [36]. The survival advantage of Pseudomonadota in the oral cavity of healthy dogs indicates its adaptation to the oxygen-rich environment and its role in maintaining oral microecological balance. As an anaerobic environment develops, it becomes more conducive to the growth of anaerobic bacteria, which gradually accumulate, compete for oral ecological niches, and become dominant. As the disease progresses, Bacillota and Bacteroidota increase at the phylum level alongside a decrease in Pseudomonadota. The negative correlation among these phyla underpins the significant changes observed in canine PD at the phylum level.

*Porphyromonas* was identified as a dominant marker genus in the oral microbiome of dogs with plaque. This suggests that *Porphyromonas* may play a crucial role in the formation and maturation of dental plaque biofilm, further promoting calculus deposition and the progress of PD. *Porphyromonas* can modulate the host’s innate immune response, leading to the overexpression of interleukins and cyclooxygenase-2, which are directly associated with periodontal tissue damage [37]. *Porphyromonas* is considered a potential pathogen correlated with the severity of PD [32,34,35,38,39].

The relative abundance of *Bacteroides* was low both in the H and P groups, but increased significantly in the C group. This increase may be attributed to alterations in the oral environment accompanying plaque and calculus formation. As the biofilm matures and mineralizes, microbial dysbiosis occurs: beneficial bacterial populations decline, whereas pathogenic taxa increase. These pathogenic microorganisms produce enzymes and bacterial toxins that act as harmful metabolic by-products, intensifying the host immune response. The ensuing chronic inflammation leads to gingival tissue destruction and the accumulation of protein-rich gingival crevicular fluid within the anaerobic periodontal pocket. Such an environment may favor the proliferation of proteolytic anaerobic bacteria like *Bacteroides* in advanced disease, providing further evidence of the association between PD and increased *Bacteroides* abundance [32].

Both *Conchiformibius* and *Pasteurellaceae*, which belong to the Pseudomonadota, accounted for the primary proportion in the oral microbiome of healthy dogs. These taxa may act as commensal bacteria within the oral microbial community and contribute to maintaining oral microbial homeostasis. *Conchiformibius* could serve as a biomarker species for the H group, indicating that its presence may be associated with favorable oral health, which is consistent with the observations reported by Watanabe et al. [38]. As oral health deteriorated, the proportions of both *Conchiformibius* and *Pasteurellaceae* decreased, suggesting that these taxa may be poorly adapted to the environmental shifts associated with dental calculus formation.

The relative abundance of *Moraxella* and *Campylobacter* increased in the P group but decreased in the C group. In particular, the relative abundance of *Moraxella* in the C group was even lower than that in the healthy group, falling below 1%. Some studies have reported that the relative abundance of *Moraxella* is negatively correlated with the scores of PD, dental plaque, calculus, and pocket depth, suggesting that *Moraxella* is associated with oral health [34,40,41]. The important role of *Moraxella* in early biofilm formation has been established [42]. It is likely due to its involvement in initial plaque development that *Moraxella* becomes the second most dominant genus in the plaque group, following *Porphyromonas*. *Campylobacter* spp. are capable of forming biofilms, promoting calculus formation and PD progression [43]. However, studies on *Campylobacter* in canine PD remain limited. *Campylobacter rectus* (formerly *Wolinella recta*) is a common member of the canine oral microbiota and possesses multiple virulence factors such as flagella, surface-layer (s-layer) proteins, rtx-type toxins, groel-like proteins, and lipopolysaccharides [44]. It is a major periodontal pathogen in dogs and acts synergistically with other anaerobic bacteria in the onset and progression of PD [4,45]. Although *Campylobacter rectus* was not detected in any of the three groups in this study, we identified an as-yet-unnamed species—*Campylobacter* sp. *RM15925*. Its relative abundance showed a similar trend to that of the *Campylobacter* genus overall, suggesting it may also act as a potential periodontal pathogen. The genus *Campylobacter* comprises numerous species, and the roles of many emerging species in PD development warrant further investigation.

Previous studies have reported that the proportion of *Porphyromonas* spp. increases with the severity of PD [32]. In the present study, *P. cangingivalis* exhibited a relatively high abundance across all three groups and was identified as a core species common to all groups. This may be attributed to its metabolic flexibility and ability to produce virulence factors, enabling it to survive and maintain a competitive advantage in both healthy and diseased oral environments [39]. A further hypothesis is that *P. cangingivalis* possesses a complete biosynthetic pathway for protoporphyrin IX. This allows it to synthesize its own heme, rather than relying on exogenous heme from blood as an energy source. This is distinct from most Porphyromonas species, which require external heme for growth. Additionally, differences in other metabolic pathways, such as the ability to synthesize vitamin B12, suggest enhanced metabolic flexibility in *P. cangingivalis*. Such versatility may contribute to its prevalence in the canine oral cavity [46]. The enrichment of *P. gingivicanis*, *P. gulae*, and *P. canoris* in the P group indicates their strong pathogenic potential. There is limited research on *P. canoris* within the canine oral flora. It may contribute to calculus formation and PD progression through interspecies competition or cooperation with other *Porphyromonas* spp. The role of *P. gulae* in PD has been extensively studied. As a black-pigmented, Gram-negative anaerobic bacterium, it is a major periodontal pathogen in dogs. It adheres to and invades periodontal tissue cells, such as periodontal ligament fibroblasts and gingival epithelial cells, which is a critical step in the pathogenesis of periodontitis [47]. The fimbrial subunit protein (FimA, approximately 41 kDa), encoded by the fimA gene, is a key virulence factor of *P. gulae*. Three FimA genotypes (A, B, and C) have been identified, all of which can be isolated from dogs with PD. Type C demonstrates the highest virulence, inducing more severe systemic inflammation and exerting the strongest inhibitory effects on cell proliferation and migration. These findings strongly support the role of *P. gulae* in impairing cellular functions, suggesting that type C FimA may serve as a marker for identifying dogs at high risk of periodontitis [48,49]. In summary, the virulence factor FimA is likely associated with the colonization of *P. gulae* in periodontal pockets and the pathogenicity of periodontitis [48]. Furthermore, both *P. gulae* and *P. gingivicanis* produce high levels of hydrogen sulfide (H_2_S) and methyl mercaptan (CH_3_SH), which may contribute to halitosis and tissue destruction in PD [50].

Previous studies have indicated that a reduction in *Conchiformibius steedae*, a potentially beneficial oral bacterium, could be associated with gingivitis and periodontitis secondary to calculus formation, as well as with increasing age [38,51]. Unlike the typical lateral division of rod-shaped bacteria, *C. steedae* has evolved a novel reproductive strategy termed multicellular longitudinal division (MuLDi). This unique adaptive mechanism may facilitate its survival under specific oral conditions [52]. It is hypothesized that changes in the oral environment—such as hypoxia and mineralization accompanying calculus formation—may disrupt its normal evolutionary adaptation, leading to a decline in its proportion. Maintaining the relative abundance of *C. steedae* could, in turns, potentially suppress the overgrowth of pathogenic bacteria and help maintain the microbial stability characteristic of oral health. Therefore, *C. steedae* may be considered as a potential candidate for the development as an oral probiotic aimed at preventing PD.

*Peptostreptococcus canis*, *Bacteroides pyogenes*, *Desulfomicrobium orale*, and *Desulfovibrio* sp. *OH1209 COT-279* exhibited substantial increases in the C group, all of which are capable of producing H_2_S. Previous studies have indicated that *Peptostreptococcus* spp. and *Bacteroides* spp. exhibit particularly active H_2_S production capabilities [53]. *Peptostreptococcus canis* is a late colonizer frequently detected in severe PD [54]. Its enrichment in the C group may suggest that certain dogs with CI ≥ 2 have developed into more severe periodontitis. *Desulfomicrobium orale* and *Desulfovibrio* sp. *OH1209 COT-279* belong to sulfate-reducing bacteria (SRB). SRB are anaerobic microorganisms that constitute part of the normal oral microbiota and prefer to thrive in the anaerobic environment of periodontal pockets. They contribute to the mineralization of fermentation products and act as terminal degraders in the anaerobic breakdown of organic matter [55,56,57]. *Desulfomicrobium orale* was first isolated from the subgingival plaque of a periodontitis patient [58] and is frequently detected in the oral cavity of individuals with gingival issues such as bleeding gums and PD. *Desulfovibrio* spp. often dominate in various inflammatory processes, and a high abundance of Desulfovibrio is indicative of elevated H_2_S production [59]. H_2_S, a byproduct of SRB sulfate reduction, is closely linked to the progression of oral diseases. It exerts cytotoxic effects by inhibiting mitochondrial cytochrome oxidase and significantly induces apoptosis in oral cells, including human periodontal ligament cells and gingival fibroblasts [60,61]. As pathogenic bacteria accumulate, H_2_S levels increase, promoting the development of PD such as gingivitis and periodontitis through its involvement in key cellular mechanisms including oxidative stress, apoptosis, and inflammation [62]. We speculate that during dental calculus formation, the subgingival niches are progressively occupied by calculus and anaerobic environment intensify. The relative abundances of anaerobic SRB in the oral microbiome are gradually increased, leading to elevated H_2_S production and deepening of periodontal pockets. All these changes further accelerate PD progression. Future research on the oral microbiome should place greater emphasis on H_2_S-producing bacteria, as they may enrich the list of bacterial species that serve as indicators for the onset or progression of PD.

According to the functional prediction results from PICRUSt2, compared to healthy dogs, the relative abundance of oral microbiota involved in the ABC transporters pathway was significantly lower in dogs with dental calculus. ABC transporters are involved in the transport of various substances, including the uptake of nutrients and metal ions, secretion of peptides, lipids, polysaccharides, and proteins, as well as excretion of toxins, inorganic ions, drugs, and antibiotics [63]. We speculate that in a healthy state, the oral microbiota sustains a robust immune response, supporting a high level of ABC transporter-dependent symbiotic bacteria. With calculus formation, inflammatory stimuli and a mineralized environment may suppress certain ABC transporter functions. Coupled with changes in microbial composition, the relative abundance of pathogens that are less dependent on ABC transporters may increase, leading to a decline in ABC transporter-related metabolic activity in dogs with calculus. This decline may result in accumulation of bacterial toxins and other harmful metabolites, further damaging periodontal tissues and creating a vicious cycle.

In summary, significant differences in the species composition of the canine oral microbiota were observed throughout the process from a healthy oral state to the formation, development, and maturation of early dental plaque, and ultimately to mineralization into dental calculus. We found that the relative abundance of certain bacterial species positively correlated with the degree of calculus formation. These species may represent “selectively enriched” microbiota during the mineralization process of dental plaque and could serve as indicator species for canine dental calculus. They may also be associated with the development of oral diseases such as gingivitis and periodontitis. In contrast, some species showed a negative correlation with calculus severity and may be associated with oral health. However, the mechanisms underlying the roles of these species in calculus formation require further investigation. The presence of H_2_S-producing bacteria in the C group may provide additional insights into the mechanisms of calculus formation and PD progression. Notably, not all species exhibited a linear increase or decrease in relative abundance across the three sample groups. Some species reached their peak or lowest proportions in the P group, posing a challenge for using the abundance trends of specific species to assist in differentiating oral health status in dogs.

As shown by the statistical characteristics of the dogs included in this study, the oral condition deteriorated and dental calculus gradually formed with increasing age, as expected. We attempted to control other variables that might influence the composition of the oral microbiota. In future studies, increasing the sample size and minimizing confounding factors could further enhance the robustness of the results. Longitudinal studies tracking the same group of dogs from health through calculus formation would also provide precise understanding the dynamic changes in the microbiome with age, diet, and other factors throughout disease progression. It should be noted that while PICRUSt2 can predict the functional profiles of 16S sequencing data, it relies on the completeness of known genomic databases, which may limit the accuracy of predictions. Furthermore, since its results are based on phylogenetic inferences, some degree of error and uncertainty is present. In cases where higher precision of functional data is required, metagenomic sequencing may still be necessary to validate the findings. Furthermore, integrating microbiome data with metabolomics or other multi-omics approaches could help reveal functional changes within the microbial community, identify potential biomarker species associated with dental calculus, and advance better oral health management in dogs.

## 5. Conclusions

By applying high-resolution, and high-accuracy TGS technology to analyze the microbial diversity of canine oral samples (particularly dental calculus specimens), we identified several oral microbiota (at the species level) that have not yet been reported or received significant attention. This broadens the perspective for future sample selection in PD research and informs the choice of sequencing technologies. Such an approach potentially facilitates the identification of previously unknown pathogens and advances the etiological study of PD.

## Figures and Tables

**Table 1:** Gingiva, plaque and calculus scoring criteria.

Evaluation Index	Scoring Criteria	Score
Gingival Index (GI)	Healthy gingiva, with no inflammation at the gingival margin and normal periodontal pocket depth (free gingiva) (<3 mm in dogs)	0
	Mild gingivitis, with slight redness and swelling at the gingival margin, but no bleeding upon probing	1
	Moderate gingivitis, with redness and swelling at the gingival margin, and bleeding upon probing	2
	Severe gingivitis, with marked redness and swelling at the gingival margin, often accompanied by ulceration, and potentially spontaneous gingival bleeding	3
Plaque Index (PI)	No visible plaque	0
	Plaque covers less than 1/3 of the buccal tooth surface	1
	Plaque covers between 1/3 and 2/3 of the buccal tooth surface	2
	Plaque covers more than 2/3 of the buccal tooth surface	3
Calculus Index (CI)	No visible calculus	0
	Calculus covers less than 1/3 of the maxillofacial tooth surface	1
	Calculus covers between 1/3 and 2/3 of the maxillofacial tooth surface; little or no subgingival plaque accumulation	2
	Calculus covers more than 2/3 of the maxillofacial tooth surface and extends below the gingival margin	3

**Table 2:** Characteristics of each group.

	Healthy Group	Plaque Group	Calculus Group	*p* Value	H vs. P	H vs. C	P vs. C
Age (months)							
Mean ± SD	10.8 ± 1.81	63.6 ± 3.92	120.2 ± 10.73	<0.001	<0.001	<0.001	<0.001
Mix-Max	8–14	58–70	107–140				
Weight (kg)							
Mean ± SD	6.27 ± 3.46	6.75 ± 4.08	8.1 ± 5.22	0.554	0.739	0.353	0.393
Mix-Max	2.9–13.5	2.9–14	3.2–17				
Sex				0.873	0.653	0.435	0.653
Males, *n* (%)	4 (40%)	5 (50%)	4 (40%)				
Females, *n* (%)	6 (60%)	5 (50%)	6 (60%)				

Age and body weight data were presented as mean ± standard deviation (SD). The Kruskal–Wallis test was applied for overall comparisons, and the Mann–Whitney U test was used for pairwise comparisons. Categorical variables were presented as counts and percentages, and the chi-square test was applied to analyze intergroup differences.

## Data Availability

Raw PacBio HiFi sequences have been uploaded to the NCBI Sequence Read Archive (SRA) (accessions SAMN52657937-SAMN52657966), under BioProject PRJNA1344925.

## Supplementary Materials

The following supporting information can be downloaded at: https://www.mdpi.com/article/10.3390/ani15223335/s1, Table S1: Metadata of all dogs included in the study; Table S2: The DNA concentration yield from each sample; Table S3: ASV table.

## Abbreviations

The following abbreviations are used in this manuscript:

PD	Periodontal disease
NGS	Next-generation sequencing
TGS	Third-generation sequencing
PacBio	Pacific biosciences
SMRT	Single molecule real-time
GI	Gingival index
PI	Plaque index
CI	Calculus index
BOP	Bleeding on probing
HiFi	High-fidelity
CCS	Circular consensus sequencing
QIIME2	Quantitative insights into microbial 2
ASVs	Amplicon sequence variants
PCoA	Principal co-ordinates analysis
LEfSe	Linear discriminant analysis effect size
LDA	Linear discriminant analysis
KEGG	Kyoto encyclopedia of genes and genomes
MuLDi	Multicellular longitudinal division
SRB	Sulfate-reducing bacteria
SCFAs	Short-chain fatty acids

## References

[1] Perazzi A., Ricci R., Contiero B., Iacopetti I. (2022). Evaluation of Salivary Biochemistry in Dogs with and without Plaque, Calculus, and Gingivitis: Preliminary Results. Animals.

[2] AlRowis R., AlMoharib H.S., AlMubarak A., Bhaskardoss J., Preethanath R.S., Anil S. (2014). Oral Fluid-Based Biomarkers in Periodontal Disease—Part 2. Gingival Crevicular Fluid. J. Int. Oral Health JIOH.

[3] Buduneli N., Kinane D.F. (2011). Host-derived Diagnostic Markers Related to Soft Tissue Destruction and Bone Degradation in Periodontitis. J. Clin. Periodontol..

[4] Kačírová J., Sondorová M., Maďari A., Styková E., Mucha R., Nemcová R., Marečáková N., Farbáková J., Maďar M. (2022). Detection of Periodontal Pathogens from Dental Plaques of Dogs with and without Periodontal Disease. Pathogens.

[5] Marsh P.D. (2006). Dental Plaque as a Biofilm and a Microbial Community—Implications for Health and Disease. BMC Oral Health.

[6] Wei Y., Dang G., Ren Z., Wan M., Wang C., Li H., Zhang T., Tay F.R., Niu L. (2024). Recent Advances in the Pathogenesis and Prevention Strategies of Dental Calculus. npj Biofilms Microbiomes.

[7] Belibasakis G.N., Manoil D. (2021). Microbial Community-Driven Etiopathogenesis of Peri-Implantitis. J. Dent. Res..

[8] Tatakis D.N., Kumar P.S. (2005). Etiology and Pathogenesis of Periodontal Diseases. Dent. Clin. N. Am..

[9] Radeerom T., Thongkorn K., Buddhachat K., Pradit W., Chomdej S., Siengdee P., Nganvongpanit K. (2018). Köpek ve Kedi Diş Taşı Mikrobiyomunun Ileri Jenerasyon Sekanslama Kullanarak Araştırılması. Kafkas Univ. Vet. Fak. Derg..

[10] Coignoul E., Cheville N. (1984). Calcified Microbial Plaque. Dental Calculus of Dogs. Am. J. Pathol..

[11] Akcalı A., Lang N.P. (2018). Dental Calculus: The Calcified Biofilm and Its Role in Disease Development. Periodontol. 2000.

[12] Zhang J., Su L., Wang Y., Deng S. (2020). Improved High-Throughput Sequencing of the Human Oral Microbiome: From Illumina to PacBio. Can. J. Infect. Dis. Med. Microbiol..

[13] Kurtz Z.D., Müller C.L., Miraldi E.R., Littman D.R., Blaser M.J., Bonneau R.A. (2015). Sparse and Compositionally Robust Inference of Microbial Ecological Networks. PLoS Comput. Biol..

[14] Costea P.I., Hildebrand F., Arumugam M., Bäckhed F., Blaser M.J., Bushman F.D., de Vos W.M., Ehrlich S.D., Fraser C.M., Hattori M. (2017). Enterotypes in the Landscape of Gut Microbial Community Composition. Nat. Microbiol..

[15] Han H., Choi Y.H., Kim S.Y., Park J.H., Chung J., Na H.S. (2024). Optimizing Microbiome Reference Databases with PacBio Full-Length 16S rRNA Sequencing for Enhanced Taxonomic Classification and Biomarker Discovery. Front. Microbiol..

[16] Buetas E., Jordán-López M., López-Roldán A., D’Auria G., Martínez-Priego L., De Marco G., Carda-Diéguez M., Mira A. (2024). Full-Length 16S rRNA Gene Sequencing by PacBio Improves Taxonomic Resolution in Human Microbiome Samples. BMC Genom..

[17] Woese C.R., Fox G.E. (1977). Phylogenetic Structure of the Prokaryotic Domain: The Primary Kingdoms. Proc. Natl. Acad. Sci. USA.

[18] Starke R., Pylro V.S., Morais D.K. (2021). 16S rRNA Gene Copy Number Normalization Does Not Provide More Reliable Conclusions in Metataxonomic Surveys. Microb. Ecol..

[19] Athanasopoulou K., Boti M.A., Adamopoulos P.G., Skourou P.C., Scorilas A. (2021). Third-Generation Sequencing: The Spearhead towards the Radical Transformation of Modern Genomics. Life.

[20] Meyer M., Stenzel U., Myles S., Prüfer K., Hofreiter M. (2007). Targeted High-Throughput Sequencing of Tagged Nucleic Acid Samples. Nucleic Acids Res..

[21] Hamady M., Walker J.J., Harris J.K., Gold N.J., Knight R. (2008). Error-Correcting Barcoded Primers for Pyrosequencing Hundreds of Samples in Multiplex. Nat. Methods.

[22] Mosher J.J., Bernberg E.L., Shevchenko O., Kan J., Kaplan L.A. (2013). Efficacy of a 3rd Generation High-Throughput Sequencing Platform for Analyses of 16S rRNA Genes from Environmental Samples. J. Microbiol. Methods.

[23] Mosher J.J., Bowman B., Bernberg E.L., Shevchenko O., Kan J., Korlach J., Kaplan L.A. (2014). Improved Performance of the PacBio SMRT Technology for 16S rDNA Sequencing. J. Microbiol. Methods.

[24] Wang Y., Zhang J., Chen X., Jiang W., Wang S., Xu L., Tu Y., Zheng P., Wang Y., Lin X. (2017). Profiling of Oral Microbiota in Early Childhood Caries Using Single-Molecule Real-Time Sequencing. Front. Microbiol..

[25] Callahan B.J., Wong J., Heiner C., Oh S., Theriot C.M., Gulati A.S., McGill S.K., Dougherty M.K. (2019). High-Throughput Amplicon Sequencing of the Full-Length 16S rRNA Gene with Single-Nucleotide Resolution. Nucleic Acids Res..

[26] Guo H., Li B., Yao H., Liu D., Chen R., Zhou S., Ji Y., Zeng L., Du M. (2023). Profiling the Oral Microbiomes in Patients with Alzheimer’s Disease. Oral Dis..

[27] Ma G., Qiao Y., Shi H., Zhou J., Li Y. (2022). Comparison of the Oral Microbiota Structure among People from the Same Ethnic Group Living in Different Environments. Biomed Res. Int..

[28] Löe H., Silness J. (1963). Periodontal Disease in Pregnancy I. Prevalence and Severity. Acta Odontol. Scand..

[29] Dale S. (2007). Scherl.; Lori Coffman.; Misty Van Cleave.; Steve Lowry. Validation of a New Dental Plaque Quantification Method in Dogs. J. Vet. Dent..

[30] Ramfjord S.P. (1959). Indices for Prevalence and Incidence of Periodontal Disease. J. Periodontol..

[31] Li X., Zhao Z., Guo S., Yang C., Gao Y., Li L., Ning K., Zhang Q., Zhou N., Zhang H. (2024). Effects of Toothpaste Containing Inactivated Lacticaseibacillus Paracasei Probio-01 on Plaque-Induced Gingivitis and Dental Plaque Microbiota. Microb. Pathogen..

[32] Santibáñez R., Rodríguez-Salas C., Flores-Yáñez C., Garrido D., Thomson P. (2021). Assessment of Changes in the Oral Microbiome That Occur in Dogs with Periodontal Disease. Vet. Sci..

[33] Ruparell A., Inui T., Staunton R., Wallis C., Deusch O., Holcombe L.J. (2020). The Canine Oral Microbiome: Variation in Bacterial Populations across Different Niches. BMC Microbiol..

[34] Davis I.J., Wallis C., Deusch O., Colyer A., Milella L., Loman N., Harris S. (2013). A Cross-Sectional Survey of Bacterial Species in Plaque from Client Owned Dogs with Healthy Gingiva, Gingivitis or Mild Periodontitis. PLoS ONE.

[35] Wallis C., Milella L., Colyer A., O’Flynn C., Harris S., Holcombe L.J. (2021). Subgingival Microbiota of Dogs with Healthy Gingiva or Early Periodontal Disease from Different Geographical Locations. BMC Vet. Res..

[36] Pereira A.M., Clemente A. (2021). Dogs’ Microbiome from Tip to Toe. Top. Companion Anim. Med..

[37] Nomura R., Inaba H., Yasuda H., Shirai M., Kato Y., Murakami M., Iwashita N., Shirahata S., Yoshida S., Matayoshi S. (2020). Inhibition of *Porphyromonas gulae* and Periodontal Disease in Dogs by a Combination of Clindamycin and Interferon Alpha. Sci. Rep..

[38] Watanabe A., Okada J., Niwa R., Inui Y., Ito K., Shimokawa Y., Kihira M. (2023). Bacterial Composition Changes in Canine Plaque over Periodontal Disease Severity and Daily Care Practices. Biorxiv.

[39] Niemiec B.A., Gawor J., Tang S., Prem A., Krumbeck J.A. (2022). The Bacteriome of the Oral Cavity in Healthy Dogs and Dogs with Periodontal Disease. Am. J. Vet. Res..

[40] Oba P.M., Carroll M.Q., Alexander C., Valentine H., Somrak A.J., Keating S.C.J., Sage A.M., Swanson K.S. (2021). Microbiota Populations in Supragingival Plaque, Subgingival Plaque, and Saliva Habitats of Adult Dogs. Anim. Microbiome.

[41] Wallis C., Colyer A., Holcombe L.J. (2025). Bacterial Associations with Periodontal Disease in Yorkshire Terriers. BMC Vet. Res..

[42] Holcombe L.J., Patel N., Colyer A., Deusch O., O’Flynn C., Harris S. (2014). Early Canine Plaque Biofilms: Characterization of Key Bacterial Interactions Involved in Initial Colonization of Enamel. PLoS ONE.

[43] Man S.M. (2011). The Clinical Importance of Emerging Campylobacter Species. Nat. Rev. Gastroenterol. Hepatol..

[44] Arce R.M., Diaz P.I., Barros S.P., Galloway P., Bobetsis Y., Threadgill D., Offenbacher S. (2010). Characterization of the Invasive and Inflammatory Traits of Oral Campylobacter Rectus in a Murine Model of Fetoplacental Growth Restriction and in Trophoblast Cultures. J. Reprod. Immunol..

[45] Kato Y., Shirai M., Murakami M., Mizusawa T., Hagimoto A., Wada K., Nomura R., Nakano K., Ooshima T., Asai F. (2011). Molecular Detection of Human Periodontal Pathogens in Oral Swab Specimens from Dogs in Japan. J. Vet. Dent..

[46] O’Flynn C., Deusch O., Darling A.E., Eisen J.A., Wallis C., Davis I.J., Harris S.J. (2015). Comparative Genomics of the Genus *Porphyromonas* Identifies Adaptations for Heme Synthesis within the Prevalent Canine Oral Species *Porphyromonas cangingivalis*. Genome Biol. Evol..

[47] Iwashita N., Nomura R., Shirai M., Kato Y., Murakami M., Matayoshi S., Kadota T., Shirahata S., Ohzeki L., Arai N. (2019). Identification and Molecular Characterization of *Porphyromonas gulae* fimA Types among Cat Isolates. Vet. Microbiol..

[48] Hamada N., Takahashi Y., Watanabe K., Kumada H., Oishi Y., Umemoto T. (2008). Molecular and Antigenic Similarities of the Fimbrial Major Components between *Porphyromonas gulae* and *P. Gingivalis*. Vet. Microbiol..

[49] Yamasaki Y., Nomura R., Nakano K., Inaba H., Kuboniwa M., Hirai N., Shirai M., Kato Y., Murakami M., Naka S. (2012). Distribution and Molecular Characterization of *Porphyromonas gulae* Carrying a New fimA Genotype. Vet. Microbiol..

[50] Ito N., Itoh N., Kameshima S. (2023). Volatile Sulfur Compounds Produced by the Anaerobic Bacteria *Porphyromonas* spp. Isolated from the Oral Cavities of Dogs. Vet. Sci..

[51] Kislik G., Zhou L., Rubbi L., Pellegrini M. (2024). Age-Correlated Changes in the Canine Oral Microbiome. Front. Microbiol..

[52] Nyongesa S., Weber P.M., Bernet È., Pulido F., Nieves C., Nieckarz M., Delaby M., Viehboeck T., Krause N., Rivera-Millot A. (2022). Evolution of Longitudinal Division in Multicellular Bacteria of the Neisseriaceae Family. Nat. Commun..

[53] Persson S., Edlund M., Claesson R., Carlsson J. (1990). The Formation of Hydrogen Sulfide and Methyl Mercaptan by Oral Bacteria. Oral Microbiol. Immunol..

[54] Polkowska I., Sobczyńska-Rak A., Gołyńska M. (2014). Analysis of Gingival Pocket Microflora and Biochemical Blood Parameters in Dogs Suffering from Periodontal Disease. In Vivo.

[55] Widdel F., Zehnder A.J.B. (1988). Microbiology and Ecology of Sulphateand Sulpur Reducing Bacteria. Biology of Anaerobic Microorganisms.

[56] Hansen T.A., Odom J.M., Singleton R. (1993). Carbon Metabolism of Sulfate-Reducing Bacteria. The Sulfate-reducing Bacteria: Contemporary Perspectives.

[57] Kushkevych I., Coufalová M., Vítězová M., Rittmann S.K.-M.R. (2020). Sulfate-Reducing Bacteria of the Oral Cavity and Their Relation with Periodontitis—Recent Advances. J. Clin. Med..

[58] Langendijk P.S., Kulik E.M., Sandmeier H., Meyer J., Van Der Hoeven J.S. (2001). Isolation of *Desulfomicrobium orale* sp. Nov. and Desulfovibrio Strain NY682, Oral Sulfate-Reducing Bacteria Involved in Human Periodontal Disease. Int. J. Syst. Evol. Microbiol..

[59] Kushkevych I., Dordević D., Vítězová M. (2021). Possible Synergy Effect of Hydrogen Sulfide and Acetate Produced by Sulfate-Reducing Bacteria on Inflammatory Bowel Disease Development. J. Adv. Res..

[60] Zhang J.-H., Dong Z., Chu L. (2010). Hydrogen Sulfide Induces Apoptosis in Human Periodontium Cells. J. Periodontal Res..

[61] Murata T., Yaegaki K., Qian W., Herai M., Calenic B., Imai T., Sato T., Tanaka T., Kamoda T., Ii H. (2008). Hydrogen Sulfide Induces Apoptosis in Epithelial Cells Derived from Human Gingiva. J. Breath Res..

[62] Wu D.-D., Ngowi E.E., Zhai Y.-K., Wang Y.-Z., Khan N.H., Kombo A.F., Khattak S., Li T., Ji X.-Y. (2022). Role of Hydrogen Sulfide in Oral Disease. Oxid. Med. Cell. Longev..

[63] How S.S., Nathan S., Lam S.D., Chieng S. (2025). ATP-Binding Cassette (ABC) Transporters: Structures and Roles in Bacterial Pathogenesis. J. Zhejiang Univ.-Sci. B.

